# Survival of a long-lived single island endemic, the Raso lark *Alauda razae*, in relation to age, fluctuating population and rainfall

**DOI:** 10.1038/s41598-019-55782-8

**Published:** 2019-12-20

**Authors:** E. G. Dierickx, R. A. Robinson, M. de L. Brooke

**Affiliations:** 1 Department of Zoology, University of Cambridge, Downing Street, Cambridge, CB2 3EJ United Kingdom; 2 Fauna & Flora International, The David Attenborough Building, Pembroke Street, Cambridge, CB2 3QZ United Kingdom; 3 British Trust for Ornithology, The Nunnery, Thetford, Norfolk IP24 2PU United Kingdom

**Keywords:** Conservation biology, Population dynamics

## Abstract

Estimating and understanding variation in survival rates is crucial for the management of threatened species, especially those with limited population sizes and/or restricted ranges. Using a capture-resighting dataset covering 2004–2017, we estimate adult survival in the Raso lark *Alauda razae*, a Critically Endangered single-island Cape Verdean endemic, whose population varied 25-fold during the study. Average annual adult survival was similar for males (0.813 ± 0.011) and females (0.826 ± 0.011) over the period. These values are high for a temperate passerine but not unusual for an insular tropical species like the lark. The oldest bird was recorded 13 years after first ringing. There was strong evidence that survival varied among years (between 0.57 and 0.95), being generally higher in wetter years. Survival, especially of males, was lower when the population was large, but only in drier years. Survival declined with age but there was no evidence that this decline was other than linear. High survival, even in the face of dry conditions, at least when the population is depressed, has probably contributed to the persistence of the species on its 7 km^2^ island home over several centuries.

## Introduction

Species whose distribution is confined to a single small island are inherently vulnerable to population extinction because of the risks posed by environmental and demographic stochasticity. This vulnerability is recognized in the IUCN Red List Criteria which often classify such species as Endangered, or even Critically Endangered^1^. Population changes are explained by the balance between reproduction and mortality which, in island endemics/isolated island populations, is relatively easy to quantify since the populations are closed, with zero immigration and zero (or minimal) emigration. Despite their sometimes imperilled status, island populations can, therefore, offer clearer insights into the drivers of population fluctuations.

These considerations are well exemplified by the Raso lark *Alauda razae* of the Cape Verdes archipelago, which lies some 500 km west of Senegal. Since its scientific description about 120 years ago^2^, the lark has been confined to and survived on the uninhabited 7 km^2^ islet of Raso (16°N 24°W), which is free of introduced vertebrate predators unlike all the other nine, permanently inhabited, Cape Verdean islands. It may be that these predators contributed to the lark’s disappearance from three other Cape Verdean islands: Santo Antão, São Vicente and Santa Luzia^3^. In so far as the sub-fossil record indicates a date, it seems that these disappearances were associated with the Portuguese discovery of the archipelago in 1456 and colonization six years later^3,4^.

During the second half of the 20^th^ Century, sporadic censuses suggested the Raso lark population might sometimes have fallen to as low as 20 individuals^5^. Over that time, the Cape Verdes experienced severe and sometimes prolonged droughts lasting over 10 years and resulting in the deaths of tens of thousands of people^4,6^. Presumably such multi-year droughts also affected Raso, and severely limited the lark’s reproduction which is minimal in drought years^7,8^. If this is correct, the lark could only have persisted if adult survival, even in times of drought, was high. Following years of drought, the lark population can grow rapidly, doubling or tripling from one year to the next following significant rainfall through recruitment of newly-hatched individuals into the breeding population^7^ (Table 1). Knowledge of how climate, particularly extreme events, can influence survival is necessary to understand how populations might respond to climates that are becoming increasingly erratic^9,10^. We quantify, for the first time, survival in the tropical Raso lark and explore how it varies in response to hugely varying population density and rainfall levels.

**Table 1:** Male and female sample sizes, calculated as birds captured or resighted in each year of the study.

Year	Ringed males	Ringed females	All	Of which first ringed in current year*	Total population size
2004	15	11	26	26	57
2005	41	18	59	38	132
2006	57	27	84	37	140
2007	58	41	99	23	159
2008	71	42	113	8	184
2009	65	47	112	24	193
2010	98	76	174	76	486
2011	158	140	298	114	1558
2012	186	168	354	90	1546
2013	175	149	324	63	1314
2014	137	148	285	21	1170
2015	101	161	262	92	900
2016	118	151	269	53	908
2017	179	208	387	142	1561

Total population size is included in the table for comparison. *Excludes juveniles and nestlings.

Small, closed, populations may also be more susceptible to demographic fluctuations, for example in age or sex structure. In the early years of the study, the population was strongly male-biased^11^, a feature of many threatened bird populations^12^. Since in the case of the Raso lark the bias is very unlikely to be caused by sex differences in dispersal, and there is no reason to suppose a sex bias in primary sex ratio (own unpublished data), a sex ratio bias among adult larks suggests higher survival of males. Our dataset allows investigation of whether any sex difference in survival varies over time, which may in turn contribute to understanding the causes of such survival differences. Finally, the fact that survival is high, and the birds long-lived, allows us to assess changes in survivorship with age.

## Results

**Figure 1:**
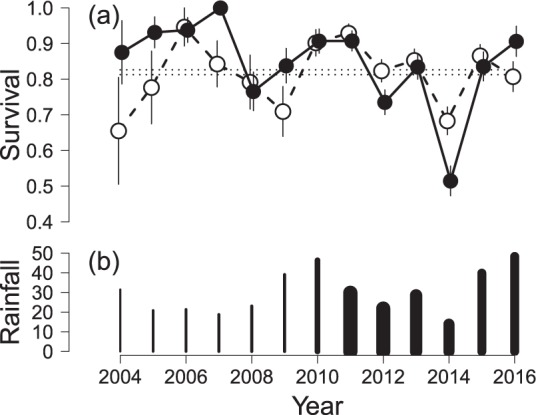
(**a**) Survival estimates for males (solid line, filled points) and females (dashed line, open points) for the time period 2004–2017, based on the model ϕ_sex*year_. Error bars indicate ±1 s.e. and the dotted lines mean survival of females (upper) and males (lower). (**b**) Mean daily rainfall (mm) for Aug-Nov immediately preceding each field season; bar widths are proportional to total population size.

The average resighting probability was high, for both females (0.87 ± 0.01) and males (0.89 ± 0.01). Average annual survival probabilities were similar between the sexes (female: ϕ = 0.826 ± 0.011; male: ϕ = 0.813 ± 0.011), but there was substantial variation between years (Fig. 1). Annual survival probabilities were more similar in the latter half of the study, when population size was higher, and they were influenced by the number of birds in the population and by rainfall.

**Figure 2:**
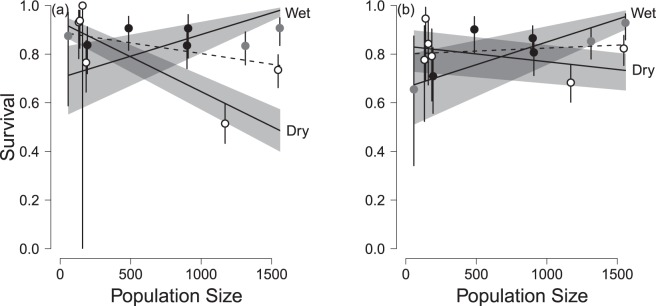
Survival of (**a**) male and (**b**) female Raso larks in relation to population size, according to whether the year was one of the six driest (open circles), three intermediate (grey dots) or four wettest (black dots); bars indicate ± 1 s.e from model ϕ_sex*PopSize + sex*Rain + sex*PopSize*Rain_. The dotted line indicates the response of survival to population size without accounting for rainfall (from model ϕ_sex*PopSize_). Shading indicates 95% confidence limits about the regression.

Rainfall alone accounted for ~23% of this variation (Table 2), with survival being higher following years of greater precipitation (β = 0.22 ± 0.04), and survival of males being greater in wetter years (β = 0.32 ± 0.06) than females (β = 0.13 ± 0.05). Population size, by itself, explained less of the variation in survival probabilities (~13%), although again the effect was strongly sex-specific with males (β = −0.00062 ± 0.00015) having lower survival in years with high population size, while females were seemingly not affected (β = 0.00017 ± 0.00016). As a result, in the nine years without strong juvenile recruitment (i.e. excluding 2004/5, 2009/10, 2010/11 and 2016/17; Table 1), annual population change was more closely related to male (r = 0.86, p = 0.003) than female survival (r = 0.54, p = 0.13). The impact of population size on survival probabilities was most marked in dry years (Fig. 2); in combination, rainfall and population size accounted for about two-thirds of the annual variation in survival probability in the population (Table 2).

**Table 2:** Models of annual variation in survival of Raso larks (2004–2017, n = 732).

Model	Npar	Deviance	ΔAIC	Rel. Dev.
sex * year	**30**	**3478**.**3**	**0**	**1**
sex + year	18	3507.5	4.72	0.816
year	17	3509.8	5.00	0.800
sex + sex:PopSize + sex:Rain + sex:PopSize:Rain	12	3532.6	17.6	0.657
sex + sex: PopSize + sex:Rain + PopSize:Rain	11	3536.0	19.7	0.631
sex + PopSize + Rain + PopSize:Rain	9	3553.5	32.5	0.525
sex + Rain + sex:Rain	8	3594.6	71.5	0.265
sex + PopSize + sex:PopSize	8	3611.7	93.3	0.128
sex + Rain	7	3600.4	75.3	0.229
sex + PopSize	7	3629.5	104	0.045
sex	6	3636.6	110	0

For each modelled set of covariates is given the number of parameters (Npar), the total deviance explained, AIC relative to the best model (ΔAIC), and the deviance relative to the full (ϕ_sex*year_) and null (ϕ_sex_) models (Rel. Dev.). Capitalised parameters are linear, those in lower-case factors (with 2, sex, or 13, year, levels). Models are listed in descending order of complexity and all include four re-encounter parameters (p_td*sex_, see text).

**Figure 3:**
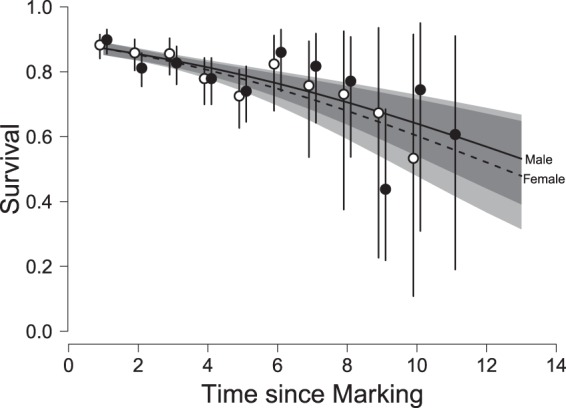
Modelled annual survival (±1 s.e.) of male (solid line, filled points) and female (dashed line, open points) Raso larks according to time since marking (as a proxy for age). Estimates are for birds without claw damage; those exhibiting damaged claws had a lower intercept (β = −0.35 ± 0.14). Shading indicates 95% confidence limits about the regression. Reading from the top, the four grey-shade boundaries represent male upper confidence limit, female upper confidence limit, male lower confidence limit, and female lower confidence limit.

Individual survival probabilities declined with time since marking (Fig. 3), and presumed older birds (those with damaged claws on ringing) had a lower survival probability (Table 3). The rate of decline in survival with time since marking was higher for females (β = −0.15 ± 0.05) than for males (β = −0.10 ± 0.04), and female individuals with damaged claws consequently exhibited a greater lowering of survival (β = −0.39 ± 0.20) compared to those with undamaged claws than did their male counterparts (β = −0.26 ± 0.19), though the estimated difference was small. The decline in survival with time since marking among the claw-damaged birds was slightly higher than among (presumed) younger individuals (β = −0.05 ± 0.07), suggesting the possibility of an accelerated decline with increasing age, but including a quadratic TSM term in the model did not improve the fit (Table 3).

**Table 3:** Variation in survival among adult Raso larks (n = 732) with time since marking (TSM, a proxy for age) and the presence of damaged claws (indicating greater age).

Model	Npar	Deviance	ΔAIC
sex + year + sex:TSM + sex:claw	22	3479.1	3.07
sex + year + sex:TSM + claw	21	3479.2	1.17
sex + year + TSM + claw + claw:TSM	21	3479.6	1.56
sex + year + TSM + claw	**20**	**3480**.**1**	**0**
sex + year + sex:TSM	20	3490.1	10.0
sex + year + TSM + (TSM)^2^	20	3490.6	10.5
sex + year + TSM	19	3490.6	8.52
sex + year	18	3507.5	23.4

For each modelled set of covariates is given the number of parameters (Npar), the total deviance explained and AIC relative to the best model (highlighted). Capitalised parameters are linear, those in lower-case factors (with 2, sex and claw, or 13, year, levels). Models are listed in descending order of complexity and all include four re-encounter parameters (p_td*sex_, see text).

An estimate of the survival of individuals of known age indicated that survival of first-year birds (ϕ = 0.63 ± 0.05) was lower than that of adults, and supported a decline in survival with age (Table 4), although the estimate of the rate of decline was small (β = −0.01 ± 0.17).

**Table 4:** Survival in relation to age for birds of known age (those ringed as nestlings or juveniles, n = 124).

Model	Npar	Deviance	ΔAIC
year + age	28	656.3	23.1
year + Age	17	662.2	4.33
year	**15**	**662**.**2**	**0**

For each modelled set of covariates is given the number of parameters (Npar), the total deviance explained and AIC relative to the best model (highlighted). Capitalised parameters are linear, those in lower-case factors. Models are listed in descending order of complexity and all include four re-encounter parameters (p_td*sex_, see text).

Despite the marked sexual dimorphism, there was no discernible effect of bill or wing length on annual survival of males and females, and that remained true regardless of rainfall (Table S1).

## Discussion

Using a capture-resighting dataset of colour-ringed individuals observed from 2004 until 2017, we show that annual survival of adult Raso larks is generally high, but fluctuates in response to a combination of population density and rainfall. Additionally, survival was age-related, with older birds having lower survival rates, especially amongst females.

Previous studies (usually of insectivorous passerines) have shown that survival may be increased in wetter^13,14^ or drier^15^ times. Survival in the Raso lark, primarily a granivore during drier periods, was lower in drier years, in common with other granivores living in arid environments^16,17^, suggesting food resources may be a critical mediating factor^18^. Since rainfall also promotes breeding activity and larger clutches^8^, the implication is that there is little or no trade-off between reproduction and survival^19,20^ in this species. Instead, the Raso lark seems to elevate its reproductive effort to take advantage of wetter years with more resources, avoiding this trade-off. Moreover, the impact of rainfall per se on survival was more pronounced than that of population size, which nonetheless interacted as a factor with rainfall: in wetter years, survival was little affected by population size despite an order of magnitude variation in population size. The absence of a signal of density-dependence in these wetter years was unexpected. However, in drier years, when food was presumably scarcer, survival was depressed when the population was higher.

This study bears on two discussion strands concerning avian survival. The first focuses on the characteristics of islands and the distinct impact that they could have on the survival rates of island species. Researchers frequently describe a pattern whereby island species and island populations of widespread species have higher survival rates than their continental counterparts^21,22^. Compared to continental birds, island species may shift more resources towards self-maintenance as a means of increasing survival in order to maximize lifetime reproductive success; they may be able to do this because of the generally more stable, milder climates on islands and the lower prevalence of parasites and predators^22^. These arguments are applicable to the island of Raso, which has a fairly stable temperature, little annual variation in daylength, very few predators for the lark and, in all likelihood given its aridity, few lark parasites^23^.

The Raso lark’s annual adult survival rate is certainly high compared to the majority of continental passerine bird species. Its closest relative, the skylark *Alauda arvensis*, has a trans-Palaearctic breeding distribution and a much lower annual survival rate, estimated by different researchers between 0.39 and 0.78, with most studies placing it around 0.50–0.60^24^. The Raso lark’s survival rate is also higher than that of the continental Dupont’s lark *Chersophilus duponti*^25^, and much higher than that of most other continental passerines. Blake and Loiselle^26^ estimate average survival rates of forest species in Eastern Ecuador at 0.58. Peach *et al*.^27^ estimate average survival rates of granivorous southern African passerines at 0.54, and of insectivorous and nectarivorous passerines at 0.72. In Nigeria, McGregor *et al*.^28^ estimate the average survival rate of birds at 0.60. Another survey reported a mean survival rate of 0.53 in North American passerines^29^. However, island passerines seem generally to have higher survival rates, comparable to that of the Raso lark^21,30–32^.

The second (highly debated) conjecture is that tropical passerines have higher survival rates than their temperate counterparts^26,29,33^. This conjecture is based on the fact that birds in the tropics generally lay smaller clutches than in the temperate zones, and on the inverse relationship between fecundity and survival^29^. It is doubtful whether this argument can be applied to the Raso lark whose survival (this study) and fecundity^8^ are both higher when rainfall is higher.

Although average survival of males and females was similar, the most supported model (Table 2) includes an interaction term between year and sex, suggesting that the influence of sex on adult survival varies over time. Indeed, while males seem to have had higher survival at the beginning of the study, this trend reversed between 2011 and 2015 (Fig. 1). Coupled with the population expansion derived from the recruitment of equal numbers of young males and females to the population, the reversal abolished the sex ratio bias that so prominently favoured males in the early years of the study^11^ (Table 1). One potential factor explaining variation in the relative survival of males and females, body size, had no discernible effect (Table S1).

Interestingly, survival of females was less influenced by both of the environmental factors we explored (rainfall and population size) than that of males. A similar result has been shown recently in a Neotropical wren, indicating that another limiting factor can over-ride local environmental effects, perhaps through the increased cost of reproduction incurred by females^34^. Consistent with this, the survival of females declined more with age than did that of males (Fig. 3). This implies that if there is a sustained period of low reproduction, then a male bias (as was observed in the early years of the study; Table 1) will arise demographically if recruitment is insufficient to counterbalance the higher female mortality. Thus, these results suggest that the male bias commonly reported in threatened populations^12^ may result from sustained low reproductive success rather than, necessarily, being induced directly by some environmental factors affecting female survival, at least in longer-lived species.

These results also suggest a possible explanation for why male survival was lower than that of females during 2011–2015. This could be a direct consequence of the reduction of the survival of males, but not of females, caused by the large population then on the island. Why the high population size should differentially impact males is not clear: one possibility is that their larger body size and metabolic needs place them at a disadvantage compared to smaller females when the population is high and competition for resources presumably most intense (although there was no evidence of differential survival between smaller and larger individuals). The phenomenon is unlikely to be caused by the cost of intrasexual competition for mates or territorial defence in males, as when the population is large and the environment is dry, birds generally forgo breeding and territory defence, and instead forage in flocks.

Older birds experienced lower survival than younger birds, a finding replicated in a number of other passerine studies^35–37^. Despite the fair size of our dataset, it was not possible to determine whether the decline in survival was more or less linear or whether there was an age at which the decline in survival with age abruptly steepened^38,39^. Anecdotally, the fact that five birds have been observed 12 years after first capture and only one subsequently at 13 years suggests that this could be an age at which survival probabilities deteriorate sharply.

## Conclusion

The Raso lark is an island-dwelling tropical species, typically with high survival. Only when a very dry year (or years) coincides with a high population is survival substantially depressed. These features enable the species to persist through several years of drought, investing in survival and only making the additional investment in reproduction when conditions once more become relatively favourable^40^. Such a strategy is viable because, at least for the first ten years of life, senescence approaches slowly. The features also clarify how the species has survived for several centuries on a single small island with a population at times falling below 100. Additionally, this suggests that, in this and similar species, conservation management actions may best target reproductive potential or, indeed, increase the range of the species, if this has contracted historically. With this in mind, and the Raso lark facing an uncertain future, we are currently attempting to (re-) establish a second population on Santa Luzia^41^.

## Methods

### Fieldwork on raso

After trial visits in 2002 and 2003, the present study commenced in 2004 and has continued annually to 2017 with single visits each year lasting 12–20 days. These visits occurred in November or early December. This is towards the end of the period, August-November, when birds are most likely to be breeding following rainfall^8^. However breeding is rain-dependent and may occur at other times of year, rain permitting. This fieldwork schedule meant we encountered numerous breeding attempts but have no data on the number of attempts individual birds may make in a year.

All birds were captured and ringed under permits issued by the Direcção Nacional do Ambiente (Environment Ministry), Cape Verdes. This catching was done by M. de L. B., a fully-licensed bird ringer (British Trust for Ornithology permit A 1871 MP). Thanks to the species’ approachability, Raso larks can be captured individually by two people carrying a mist net, fully extended and horizontal, on two poles. When a target bird is sighted, it is approached downwind and the net dropped over the bird, which is then extracted immediately. Each bird received an individually-numbered metal ring and a unique combination of three Darvic colour rings. During the 2–3 week visit to Raso in November or early December, the 2-person team caught and ringed new flying birds, recorded the colour-ring combinations of surviving birds ringed in previous years, and also ringed nestlings and juveniles (<3 months old). The latter were readily recognized by their browner plumage with broader pale feather edgings.

Towards the end of each year’s visit, the sustained reading of colour-rings attached in previous years consistently generated resighting rates approaching 90% (see Results). We therefore knew with fair accuracy the number of colour-ringed birds on the island, the sum of those ringed in the current year plus those ringed in previous years. The number of colour-ringed birds was corrected the following year to account for the small number (usually 15–20) of colour-ringed individuals that were spotted then but had been missed in the previous year, thus accounting for the incomplete detection of individuals. This correction is applied to the population values presented by Brooke^7^ that are used in our analyses. Transects conducted across the island then allowed an estimate of the proportion of birds that were colour-ringed, from which we calculated an overall population estimate. The population has varied greatly in size during the study: in the early years (until 2009), the population did not exceed 200 individuals, with a minimum of 57 recorded in 2004. After a dramatic increase from about 200 in 2009 to 1550 in 2011, the population fluctuated until 2017 but did not fall below 900 individuals (Table 1).

### Survival modelling

Survival of birds over the period 2004–2017 was estimated by fitting Cormack-Jolly-Seber (CJS) models to datasets with marked individuals^42^ using RMark^43,44^. Initial goodness-of-fit testing (using Program RELEASE) suggested that the data fitted the CJS model acceptably (χ^2^_41_ = 55.8, p = 0.062), but closer inspection revealed evidence of significant ‘trap-dependence’ (Test 2.CT: χ^2^_10_ = 30.1, p < 0.001) with males, in particular, being more likely to be re-encountered if they had been seen the previous year. Consequently, we model resighting probabilities as sex-specific and dependent on whether the individual had been encountered in the previous year or not (i.e. four parameters). Including a time component in the resighting model did not change the survival estimates substantially. During the field period, an attempt was made to find all colour-ringed birds present on the island over a similar length of time, so we assume equal resighting rates across years. Our notation follows that of Lebreton *et al*.^42^, with a ‘*’ indicating an interaction between covariates and a ‘+’ indicating that covariates are additive (i.e. that they vary in parallel); also we use capitalised names to denote linear covariates and lower case to denote factorial covariates (thus, in our study, ‘Year’ explains 1 d.f. and ‘year’ 13 d.f.).

### Annual variation in survival

We were interested in the determinants of survival between years and considered three variables: sex, population density and rainfall. All post-juvenile birds (known juvenile birds are excluded from this analysis) could be sexed on size; indeed the Raso lark is one of the most sexually dimorphic lark species, with, for example, male bills 20–25 percent longer than those of females^11,45^. Rainfall has not been consistently directly measured on Raso, so we extracted an annual measure from the remote-sensed NCAR TRMM Multi-satellite Precipitation Analysis dataset (TMPA v7^46^), which is available daily at 0.25° resolution. We downloaded the monthly accumulated combined microwave-infrared data for the 5° square centred on Raso and bounded by 14°N 22°W, 19°N 27°W for the period of the study from https://pmm.gsfc.nasa.gov/data-access/downloads/trmm; this smooths out some of the stochasticity inherent in estimation at finer resolutions. On Raso, most precipitation occurs in the latter half of the calendar year so we summed daily totals for the period August – November and related these to the survival of larks for the following year, assuming survival to be related to food resources that are determined by rainfall in this period.

We were less interested in identifying the most parsimonious model (i.e. our ‘best’ estimate of annual survival), than the relative importance of the different covariates in determining the observed variation in annual survival. Consequently, following Grosbois *et al*.^47^, we calculate the amount of deviance explained relative to our null model, survival constant over time but differing by sex, and the full model, survival estimated separately for each sex in each year; this measure is then analogous to the R^2^ metric familiar from linear regression.

### Age-specific variation in survival

We looked for age-specific variation in two ways. First, with the dataset of adult captures, we looked for a relationship with time since marking (tsm), as this will correlate with age. In common with most lark species, Raso larks undergo a complete post-juvenile moult, probably when they are about three months old, after which birds of different ages cannot be distinguished, so tsm is not perfectly correlated with age as most individuals were ringed at unknown ages. It is worth noting, though, that, in years of major population expansion (e.g. a three-fold growth from 2010–2011), at least two-thirds of the birds in the population are one-year-old. In practice, the proportion of one-year-olds among newly-ringed birds will be high because older birds are often already ringed and trap-shy while the younger birds are naïve to trapping. Furthermore, while nestlings (n = c 150), juveniles (n = 22) and known one-year old birds (n = 3) never have damaged claws or toes, approximately one-third of known age birds captured when two or older do exhibit such damage to their claws. This damage is apparently never repaired^7^; therefore we can treat birds first caught with damaged claws or toes as a separate group, older on average than those with undamaged claws (although the exact extent of the age difference is not known). We test both linear and non-linear quadratic forms of time since marking (TSM, TSM^2^). Since the fully parameterised model (sex * year * tsm) was not identifiable, due to sparseness of data, we do not present R^2^ values for these age-related models of survival.

Secondly, we looked for age-specific variation in survival of a smaller set of birds marked as pulli or newly fledged juveniles (n = 131, of which 83 were re-encountered after ringing). Here, we estimated survival separately in the first-year (expecting it to be lower), with survival in subsequent years as a linear function of age. As juvenile birds cannot be sexed reliably in the field, we do not include this factor in these known-age models.

### Body size

Since the Raso lark is strongly sexually size dimorphic^11,45^, we also assessed whether body size influenced survival, independently of sex, by measuring wing length (flattened chord) and bill length from tip to base of skull. Both measurements show minimal overlap between the sexes. To minimize observer bias, all these measurements were made by M. de L. B.

## Supplementary information

Table S1
