# Supplementary material for: Survival of a long-lived single island endemic, the Raso lark Alauda razae, in relation to age, fluctuating population and rainfall
Source: Sci Rep. 2019 Dec 20;9:19557. doi: 10.1038/s41598-019-55782-8 (PMC6925214; doi:10.1038/s41598-019-55782-8)
Supplement: Supplementary file 1 — Table S1 [file 41598_2019_55782_MOESM1_ESM.docx]

SREP-19-03056 Dierickx et al. for Sci Repts Supplementary Material

**Survival of a long-lived single island endemic, the Raso lark *Alauda razae*, in relation to age, fluctuating population and rainfall.**

**E.G. Dierickx, R.A. Robinson & M. de L. Brooke**

**Supplementary Material**

**Table S1** Survival of adult Raso larks (n=701) in relation to individual wing and bill lengths. For each modelled set of covariates is given the number of parameters (Npar), the total deviance explained and AIC relative to the best model (highlighted). Capitalised parameters are linear, those in lower-case factors. Models are listed in descending order of complexity and all include four re-encounter parameters (p_td*sex_, see text).

| Model | Npar | Deviance | ΔAIC |
| --- | --- | --- | --- |
|  |  |  |  |
| sex * year | 30 | 3311.6 | 0 |
| sex * Wing | 8 | 3467.9 | 111.5 |
| sex + Wing | 7 | 3468.4 | 110.0 |
| sex * Bill | 8 | 3468.9 | 113.5 |
| sex + Bill | 7 | 3469.0 | 111.6 |
| sex | 6 | 3469.0 | 108.6 |
|  |  |  |  |
